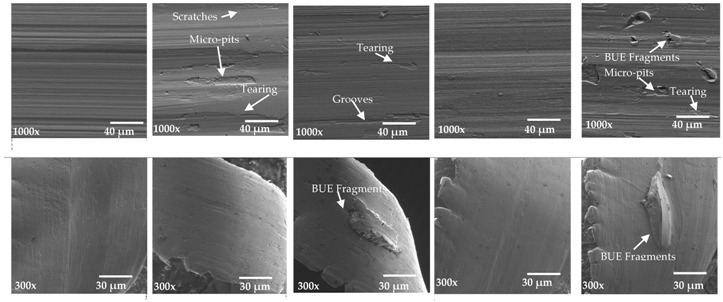# Effect of Built-Up Edge Formation during Stable State of Wear in AISI 304 Stainless Steel on Machining Performance and Surface Integrity of the Machined Part

**DOI:** 10.3390/ma10111230

**Published:** 2017-10-25

**Authors:** Yassmin Seid Ahmed, German Fox-Rabinovich, Jose Mario Paiva, Terry Wagg, Stephen Clarence Veldhuis

**Affiliations:** 1 McMaster Manufacturing Research Institute (MMRI), Department of Mechanical Engineering, McMaster University, 1280 Main Street West, Hamilton, ON L8S4L7, Canada; gfox@mcmaster.ca (G.F.-R.); paivajj@mcmaster.ca (J.M.P.); waggt@mcmaster.ca (T.W.); veldhu@mcmaster.ca (S.C.V.); 2 Department of Mechanical and Materials Science, Catholic University of Santa Catarina, Rua Visconde de Taunay, 427-Centro, Joinville-SC 89203-005, Brazil

**Keywords:** stable state of wear, built-up edge, tool wear, surface integrity, cutting forces, AISI 304 stainless steel

## Abstract

During machining of stainless steels at low cutting -speeds, workpiece material tends to adhere to the cutting tool at the tool–chip interface, forming built-up edge (BUE). BUE has a great importance in machining processes; it can significantly modify the phenomenon in the cutting zone, directly affecting the workpiece surface integrity, cutting tool forces, and chip formation. The American Iron and Steel Institute (AISI) 304 stainless steel has a high tendency to form an unstable BUE, leading to deterioration of the surface quality. Therefore, it is necessary to understand the nature of the surface integrity induced during machining operations. Although many reports have been published on the effect of tool wear during machining of AISI 304 stainless steel on surface integrity, studies on the influence of the BUE phenomenon in the stable state of wear have not been investigated so far. The main goal of the present work is to investigate the close link between the BUE formation, surface integrity and cutting forces in the stable sate of wear for uncoated cutting tool during the cutting tests of AISI 304 stainless steel. The cutting parameters were chosen to induce BUE formation during machining. X-ray diffraction (XRD) method was used for measuring superficial residual stresses of the machined surface through the stable state of wear in the cutting and feed directions. In addition, surface roughness of the machined surface was investigated using the Alicona microscope and Scanning Electron Microscopy (SEM) was used to reveal the surface distortions created during the cutting process, combined with chip undersurface analyses. The investigated BUE formation during the stable state of wear showed that the BUE can cause a significant improvement in the surface integrity and cutting forces. Moreover, it can be used to compensate for tool wear through changing the tool geometry, leading to the protection of the cutting tool from wear.

## 1. Introduction

Austenitic stainless steels are widely used in chemical industries and nuclear power industries because they have a good combination of high mechanical strength and corrosion resistance. However, the residual stresses induced by machining operations can affect their ability to withstand loading conditions such as stress concentration, corrosion, cracking, and fatigue [1,2]. Austenitic stainless steels are considered difficult-to-machine materials because of their low thermal conductivity and high strain hardening rate during cutting [3]. Their low thermal conductivity leads to heat concentration in the cutting zone that results in high localized temperatures. In addition, their high work hardening leads to high adhesion of the workpiece material to the cutting tool, resulting unstable chip and built-up edge (BUE) formation [4].

A BUE consists of highly deformed material layers, which are bonded to the tool surface and can lead to change the tool geometry and the mechanics of the process [5]. BUE is not permanently situated on the cutting edge, but periodically becomes detached, sometimes adhering to the machined surface, and sometimes to the chip [6]. The stability of a BUE as a structure is low and the breakage of it can cause cracks and damages on the tool surface. At the same time, a thin and stable BUE can be used to protect the tool from wear by reducing the friction between the cutting tool and workpiece [7]. The demand of high quality products during the production has its attention in the workpiece surface properties, especially in the surface finish and the residual stress of the machined surfaces, due to its effects in the acting of the components and reliability [8,9]. Fatigue, creep, and stress cracking lead to product failure. Therefore, it is of extreme importance to characterize the influence of the BUE formation in the surface integrity [10]. Furthermore, the high residual stress can cause deformations, accelerate phase transformations and corrosion processes and it is one of the crucial factors in the superficial quality determination. Machining of austenitic stainless steels with BUE formation is a very complex tribological phenomenon [11]. To understand the effect of BUE formation on machining forces and surface integrity, it was necessary to study its effect in the stable state of wear (steady state zone) to isolate the effect of tool wear and ensure that all conditions are the same. 

Surface integrity of a machined workpiece can be described by three main parameters: the residual stresses, the surface roughness, and the work hardening in the surface zone [12]. The residual stress is defined as the stress that exists in an elastic body after all external loads are removed [13]. Machining generally involves a large amount of plastic deformation with extremely high strain and strain rate [8]. The residual stress in the surface layer originates from three effects: mechanical effect, which leads to severe plastic deformation, thermal effect, which causes thermal plastic flow, and phase transformation. In most machining processes, the temperature does not exceed the phase transformation point, so the phase transformation can be isolated [14]. High mechanical properties and severe work hardening of austenitic stainless steels during the chip formation process, combined with low thermal conductivity, generate high cutting forces along with high localized interfacial temperatures and adhesion in the cutting zone, which are the main reasons for high level of residual stresses [15,16,17,18,19,20]. X-ray diffraction (XRD) method of residual stress determination measures the angles at which the maximum diffracted intensity takes place when a crystalline sample is subjected to X-rays [21]. The residual stresses in the surface layer are an important factor in determining the performance and fatigue life of components. Reducing residual stresses determines mainly dimensional changes in the shape and mutual position of surfaces that can change the functional conditions of the assembly to which the workpiece belongs [22]. XRD can also provide detailed information about lattice parameters of single crystals, phase, and texture. Analysis of XRD peak profiles indicated that full-width at half-maximum (FWHM) is sensitive to the variation in stress–strain accumulation in the material [20].

Few studies on surface integrity induced in machining of AISI 304 stainless steel have been performed. Xavior et al. [23] reported that high work-hardening rate, high built up edge tendency, and low thermal conductivity of AISI 304 stainless steel are the responsible for the poor surface finish and high tool wear. Moreover, Selvam et al. [24] observed that the BUE is continually growing during machining AISI 304 and digging into the workpiece and when built-up edge reaches a critical size, it breaks and welds on the machined surface, leading to increase surface roughness. Arunachalam et al. [25] reported that high tensile residual stress values were obtained, as a result of BUE deposition on the machined surface. The authors also noted that these BUE fragments deteriorated the surface integrity under loading conditions by their effect on the fatigue crack growth. Nagawaka et al. [26] found that the cutting stresses were greater than those in the longitudinal direction and the thickness on the tensile layer was found to be 150 µm. Wiesner et al. [27] reported that stresses decrease in depth direction becoming zero at 300 µm from the surface and the tensile residual stresses were found to penetrate deeper into the workpiece when the cutting speed decreases. The authors also concluded that the mechanical effect not only produces compressive residual stresses, but can also contribute to tensile residual stresses. It is also stated that the strong work hardening of the work material and a considerable increase in the microstructural defects close to the machined surface can also contribute to tensile residual stresses. Jang et al. [8] reported that the residual stresses in the circumferential direction are tensile (close to 600 MPa) and are greater than the compressive residual stresses predominant in the longitudinal direction. The circumferential residual stresses increase slightly with the cutting speed and decrease dramatically with the depth of cut. 

Few papers are available concerning the interaction of worn cutting tools on the residual stress state in the machines surface of stainless steels [8,28]. However, a detailed analysis of residual stress depth profiles in the regime of built-up edge formation in the stable state of wear has not been carried out yet. The main objective of this study is to investigate the close link between the BUE formation process and the surface integrity when machining austenitic stainless steel AISI 304. To allow evaluation and optimization of the effects of BUE formation on machined surface, hoop and axial components of residual stresses, cutting forces and chip formation in the stable state of wear have been investigated. These investigations are supported by Alicona microscope, Scanning Electron Microscopy (SEM), Energy-dispersive X-ray spectroscopy (EDS) and XRD analyses.

## 2. Experimental Procedures

### 2.1. Work Material, Cutting Tool and Cutting Parameters

**Figure 1:**
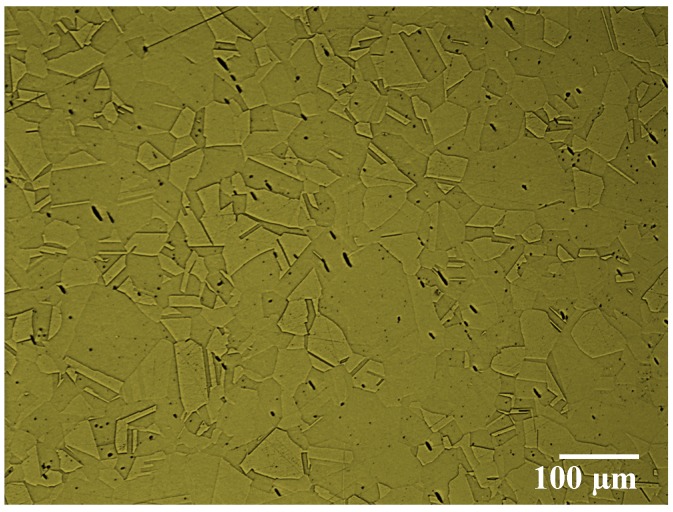
The microstructure of AISI 304 with an austenitic structure.

In this research, a round bar (120 mm diameter by 500 mm length) of an austenitic stainless steel AISI 304 was investigated during turning (finishing operation). The chemical composition and mechanical properties of the material are shown in Table 1. To reveal the workpiece microstructure, a sample of the AISI 304 was prepared, polished and etched with Glycergia solution (1 mL Glycerol + 20 mL hydrochloric Acid (HCl) + 20 mL HNO_3_). The microstructure of the AISI 304 workpiece was characterized using a Nikon ECLIPSE IV 100 microscope (Nikon Canada Inc., Mississauga, ON, Canada) equipped with UC30 camera (see Figure 1). The turning process was performed using an OKUMA CNC Crown L1060 lathe (OKUMA, Charlotte, NC, USA) with 15 kW of power and an OKUMA OSP-U10L controller. The cutting tool (Manufacturer: Kennametal) used for the experiments was an uncoated cemented carbide insert with WC/6%Co, which promotes BUE formation due to adhesion tendency to stainless steels. The designation of the insert is ISO CNGG432FS with the following geometry characteristics: back rake angle, λ_0_ = 5°; clearance angle, α_0_ = 7°; wedge angle, β = 78°; edge radius, r = 10.5 µm and nose radius, Rε = 0.8 mm. The turning tests were conducted for finishing operation with a cutting speed of 60 m/min, feed rate of 0.1 mm/rev, and depth of cut of 0.5 mm. The cutting speed v_c_ = 60 m/min was selected to ensure BUE formation. Higher cutting velocities (v_c_ > 60 m/min) lead to less provoked BUE formation, whereas lower cutting velocities (v_c_ < 60 m/min) result in wear rates that are too low for reasonable wear tests. The cutting tests were performed under wet condition to reduce friction and heat generation during the cutting process. The cutting fluid was applied at a flow rate of 11 L/min via a nozzle positioned directly above the cutting tool and directed toward the tool tip. The cutting fluid chosen was semi-synthetic coolant-CommCool™ 8800, manufactured by the Wallover Company (Harrow, ON, Canada), at a concentration of 7%, typically used with stainless steel alloys.

### 2.2. Experimental Machine Techniques

The tool flank wear was measured using a KEYENCE—VHX 5000 digital microscope (Keyence Corp., Osaka, Japan), equipped with a charge coupled device (CCD) camera and image analyzer software. The tool life criterion was set to a flank wear of 0.3 mm according to the recommendation of the ISO 3685 Standard [30]. During the tests, the cutting tools were analyzed by SEM, using a Vega 3-TESCAN (Vega 3-TESCAN, Brno, Czech Republic), coupled to EDS. The new and worn inserts were also analyzed using an Alicona Infinite Focus G5 microscope (Alicona Manufacturing Inc., Bartlett, IL, USA), which works by focus variation, to generate real 3D surface images. This microscope allows for the capture of images with a lateral resolution down to 400 nm and a vertical resolution down to 10 nm. To measure the BUE volume of a cutting edge through steady state zone, first a 3D image of a new edge of the original insert was obtained and then the edge was measured a second time after cutting. The software used the 3D image of the new edge as a reference and compared it with a 3D image of a worn edge.

To evaluate the influence of BUE formation in the steady state zone on surface roughness, the surface roughness of the machined workpiece was measured across the tool feed direction by means of an Alicona Infinite Focus-with the Profile roughness module. The procedures of surface roughness measurements were performed according to EN ISO standard 25178 [31]. Roughness measurements were taken with a cut-off wave length of 800 μm, a vertical resolution of 100 nm and a lateral resolution of 2 µm. Accuracy for roughness measurement of the microscope in terms of uncertainty was U = 25 nm at Ra = 100 nm. The surface roughness used in this study is the arithmetic mean surface roughness value (Ra), which is generally used in the industry. This evaluation was conducted three times and average reading was considered. 

**Figure 2:**
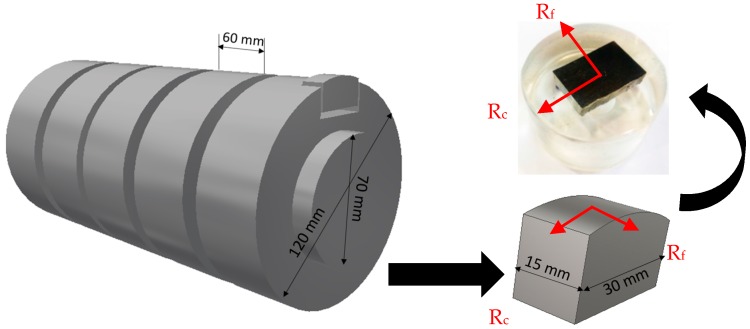
Method of sample preparation for X-ray diffraction (XRD) analysis.

Throughout the steady state region of tool wear, small pieces of the machined workpiece were cut using a water jet to evaluate any changes in machining induced residual stresses, which might be affected by BUE formation. X-ray stress data was collected using the Bruker D8 DISCOVER with DAVINCI.DESIGN diffractometer (Bruker, Burlington, ON, Canada), equipped with Cobalt Sealed Tube Source (λ_avg_ = 1.79026 Å) and Power settings: 35 kV, 45 mA. Residual stresses were analyzed in DIFFRAC.Leptos version 7.8 (Bruker, Burlington, ON, Canada), and the parameters used for analysis are summarized in Table 2. The residual stresses are measured on the machined surface and the sub-surface, in the direction of primary motion (along the cutting direction, R_c_) and the direction of feed motion (along the feed direction, R_f_) as shown in Figure 2. To determine the in depth residual stress profiles, successive layers of material with a step of 20 µm were removed by electro etching to avoid the reintroduction of additional residuals stresses [1]. In addition, in depth peak half width (FWHM) in feed and cutting direction was determined to estimate material work hardening like micro hardness [14]. 2D frames were collected with DIFFRAC.Measurement Centre Version 3.0 software (Bruker, Madison, WI, USA) integrated to 1D using DIFFRAC.EVA Version 4.0 (Bruker, Madison, WI, USA), and displayed and analyzed in Topas Version 4.2 (Bruker, Madison, WI, USA). Moreover, the machined workpiece was investigated during steady state zone using SEM to reveal the surface distortion created by BUE formation. 

During the machining tests, the cutting force measurements were performed with a 3D component tool holder Kistler dynamometer type 9121 (Kistler Instrument Corp., Amherst, NY, USA) with a data acquisition system. The signals of the forces from the dynamometer were transmitted to a Kistler 5010 type amplifier, and then recorded on a computer using LABVIEW version 14.0 software (National Instruments, Austin, TX, USA). The acquisition rate was 1000 data points per second, scale was 20 MU/volt and sensitivity was 3.85 mV/MU.

## 3. Results and Discussion

### 3.1. Investigation of the Built-up Edge in the Steady State Zone

**Figure 3:**
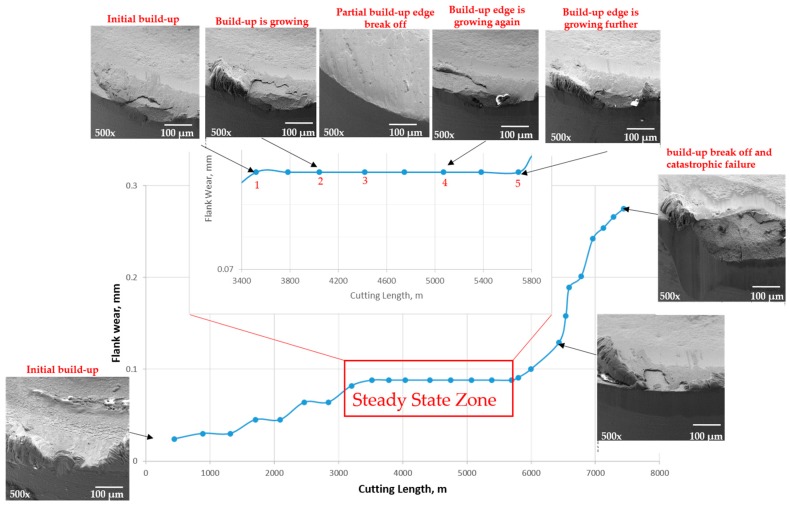
Relation between flank wear and cutting length for the uncoated carbide insert during machining AISI 304 stainless steel, combined with progressive Scanning Electron Microscopy (SEM) studies showing the Built-up edge (BUE) formation.

As discussed previously, the focus of this research is to understand the effect of BUE formation on surface integrity and cutting forces. To understand this idea in more detail, a wear performance of the uncoated cemented carbide cutting tool during machining AISI 304 stainless steel associated with progressive SEM images of BUE formation is presented in Figure 3. It can be shown that the BUE formation is unstable, and it gradually increases layer by layer and, when it has reached a certain size, it tears off. This causes cracks and damages on the tool surface and eventually results in a cutting edge break out [7]. 

The BUE increases the contact between workpiece and cutting tool and it engages the material being deformed in the same way as a punch [5]. Therefore, a large force is required to deform a material and cut the workpiece, and this force may to separate the BUE from the tool. When the BUE is not strongly attached to the tool, the friction force fractures it, and it is removed by the chip. After fracture, a new BUE is produced in the same place and the same order of events is repeated. The BUE continuously appears and disappears as long as the cutting forces increase on the tool [6]. The lack of a constant height of the BUE is characteristic of this unstable state. Therefore, the BUE changes critically the friction conditions at the tool–chip and tool–workpiece interfaces, thus affecting surface integrity of the workpiece and tool wear behavior.

**Figure 4:**
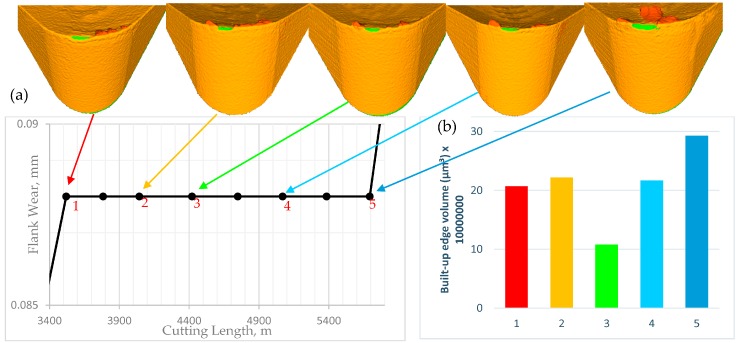
(**a**) Alicona 3D images of the cutting tool during steady state zone showing BUE propagation; and (**b**) BUE volume.

We have seen similar behavior in the steady state zone, as shown in Figure 3. The BUE is not stable at all: the build up forms, accumulates, and eventually breaks off, akin to an avalanche [7], and then a new BUE is formed again. The instability of BUE may cause cracks and damage on the tool surface and eventually leads to a cutting edge break-out [11], as it is shown in failure region (Figure 3) where a large sized build up is broken and it tears off the tool edge and results in a catastrophic failure of the cutting tool. The BUE is compressed from three sides, by the chip, the tool and the component being machined. These three forces balance each other and the BUE is held on the tool. The BUE is pressed against the tool so strongly that it sticks to it, forming a strong junction that is capable of removing chips. Under an excessive pressure, the BUE becomes so work hardened that it cut the material from which it has been produced instead of the tool and in this way it prevents the tool from wear [6]. Figure 4a shows the real 3D images of cutting tool profiles obtained with the Alicona microscope where the shapes of BUE at different cutting lengths through the steady state zone can be seen in orange and the associated values of BUE volume are shown in Figure 4b. 

**Figure 5:**
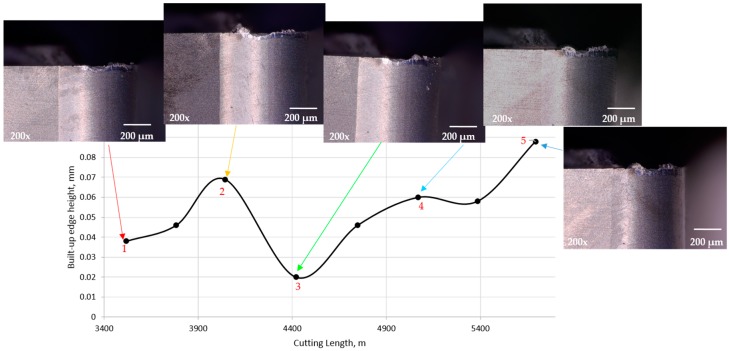
Relation between BUE height and cutting length for uncoated insert during steady state zone. Additionally the flank surface presented for some points in the steady state zone.

To understand more about how the BUE is formed through the steady state zone, it was necessary to measure its average height during cutting. The average height of BUE in the steady state zone was measured using the KEYENCE microscope and plotted in Figure 5. It is seen that gradually BUE is formed gradually for a while and when it reaches a certain magnitude, pushed by the chip along the rake face, the edge is partially uncovered, and new BUE begins to form.

**Figure 6:**
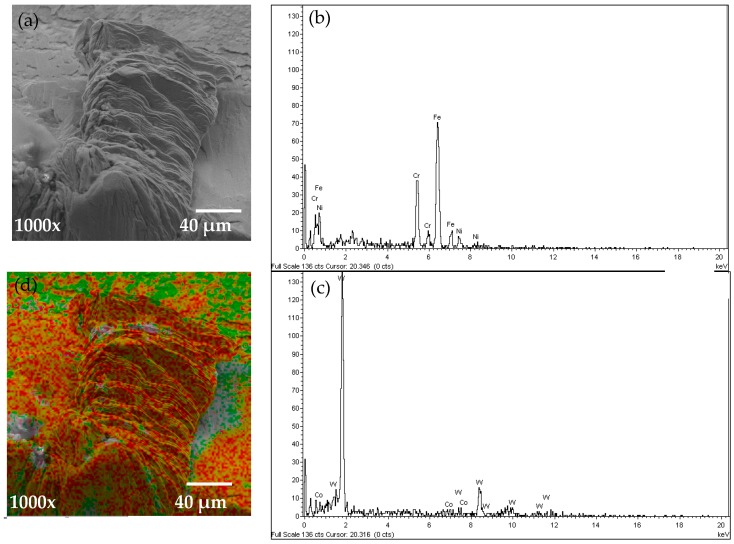
(**a**) SEM image of BUE region; (**b**) Energy-dispersive X-ray spectroscopy (EDS) spectrum analysis of the BUE zone on the rake face; (**c**) EDS spectrum analysis of the tool surface; and (**d**) color map of the Fe and Cr elements on the BUE region.

To investigate the chemical composition of the tool and BUE, an EDS analysis was performed and the results are shown in Figure 6. An SEM image of the BUE region in the steady state zone (at cutting length = 4043 m) can be seen in Figure 6a. EDS of the BUE region was performed and the spectrum illustrated in Figure 6b. The presence of elements which are not part of the composition of the tool, such as Iron, Chromium and Nickel were found, which indicates the tendency of workpiece material to cause intensive adhesion. EDS analysis of the tool surface shown in Figure 6c reveals a tungsten signal that comes from the cemented carbide substrate. The chemical composition of BUE region and the tool are given in Table 3. A color map of Iron and Chromium elements on the BUE region and tool surface are shown in Figure 6d (Fe in red and Cr in green). The EDS analysis was performed on the cutting tool for all cutting lengths in the steady state zone and similar results were observed. The influence of BUE on machining forces and surface quality are investigated in next sections. 

### 3.2. Effect of Built-up Edge Formation on Machined Surface and Chip Undersurface

**Figure 7:**
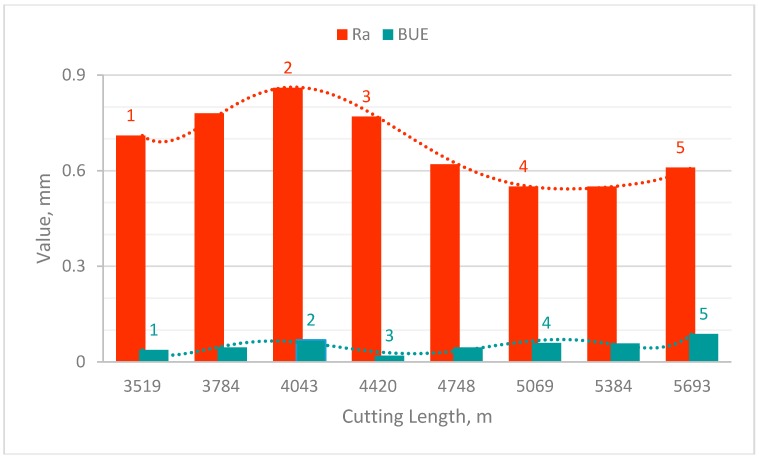
Relation between average surface roughness (Ra) and cutting length during steady state zone.

One of the main factors contributing to natural surface roughness is the occurrence of the BUE. In the steady state zone, the BUE is continually building up and breaking down, which may lead to the fractured particles being carried away on the surface of the chip and the workpiece surface. Thus, it would be expected that the larger the BUE, the rougher would be the surface produced. However another phenomenon is observed here: Figure 7 shows the average surface roughness (Ra) and BUE values in the steady state zone where it is observed that the roughness increased when BUE increases and vice versa. After 4420 m cutting length, the opposite phenomenon is observed: roughness decreased with increasing the BUE and then after 5069 m surface roughness increased with increasing BUE. To understand the main reasons for the two opposite phenomena, SEM tests were conducted on the machined surfaces and the chip undersurface. The results show in general that the machined surface presented different types of defects, such as tearing, micro-pits, grooves and scratches. It must be highlighted that these defects were less significant when the tool presented lower BUE (see points 1 and 4 in Table 4).

BUE is an unstable structure; during cutting, the highly strain hardened fragments of BUE was torn off, and may adhere to the workpiece and/or chips surface, leading to increase surface roughness. A number of different phenomena are occurring simultaneously within the steady state zone.

1. At the beginning of the steady state zone (from point 1 to point 2), BUE is continuously growing and engaging the machined surface, leading to the production of deep grooves, lowering the quality of surface finish (see point 2 in Table 4). When the BUE reaches a certain size, it tears off and new BUE is formed.

2. Before a new BUE is formed, the previous one is removed by the chip to the back of the tool rake face (Point 3 in Table 4), leading to improved surface finish.

3. Then, BUE is growing again and adheres strongly to the tool edge. Here, as shown in Point 4 in Table 4, the adhesion layer prevents sliding at the interface, protects the workpiece surface from scratching and improves the surface finish.

4. Finally, the friction force reaches a certain magnitude, overcomes the adhesion of the BUE to the tool, and plucks it off the tool. The nose of the BUE is broken off and presses into the cut surface, while the remainder is carried away by the chip. Point 5 in Table 4 shows some bright spots on both the workpiece and the chip. This confirms the proposition of Kuzentsov [6] that the bright spots on the machined surface are caused by the reminders of the fractured BUE [1].

### 3.3. Effect of Bulti-up Edge Formation on Residual Stresses and Peak Half Width

**Figure 8:**
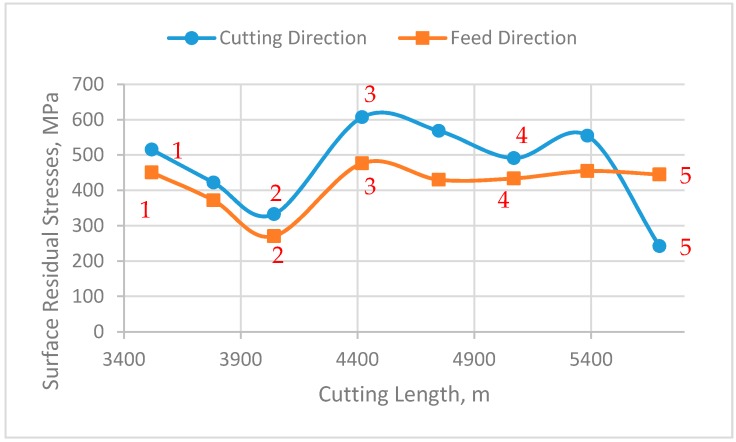
Relation between surface residual stresses and cutting length during steady state zone in cutting and feed directions.

As discussed before, the residual stresses induced are very important parameters that should be considered in the design of mechanical parts. The stresses will be tensile or compressive depending on the balance between the mechanical and thermal loads [32]. Therefore, it is necessary to understand how the BUE affects the induced residual stresses. In this section the BUE formation can be related to a gradual evolution of the friction conditions at the tool–chip interface during the chip formation process in the steady state zone, which leads to the different results for residual stress distribution (Figure 8). As observed, all values of surface residual stresses are tensile, meaning that the thermal effect is more significant than the mechanical effect, and their magnitude varied, depending on the height of BUE. As it is shown in Figure 8, tensile residual stresses decreased with increasing of BUE, which can be attributed to the high cutting temperature, leading to softening of the material, and eventually, decrease in the residual stresses [1]. Figure 8 also shows that the values of hoop stresses were higher, ranging from 240 to 550 MPa, than those of axial stresses, varying between 270 and 450 MPa. The high level of tensile residual stress both in hoop and axial directions is due to high mechanical properties and severe work hardening of austenitic stainless steel, combined with low thermal conductivity.

**Figure 9:**
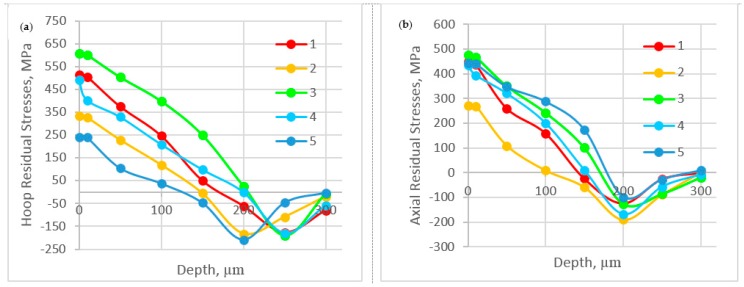
In depth residual stresses profiles for five selected points in the steady state zone in: (**a**) cutting direction; and (**b**) feed direction.

Residual stress profiles measured in the cutting and feed directions, respectively, are shown in Figure 9. It can be seen that tensile residual stresses at the near surface drop to compressive residual stresses within a shallow layer until a maximum compressive peak value is reached, and after that residual stresses are stabilized at a level corresponding to the state of the material before machining. In addition, it is observed from residual stress profiles in the cutting direction that the maximum residual stress and its depth are approximately the same for all selected points of steady state because all points have the same flank wear (stable state of wear). 

**Figure 10:**
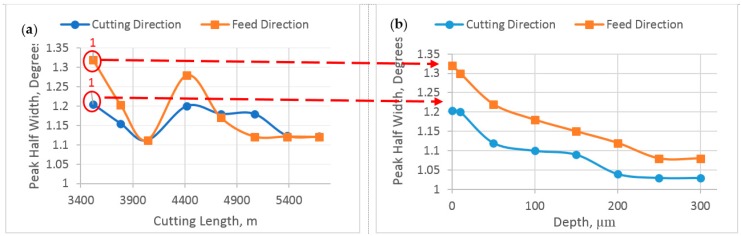
(**a**) Relation between Peak Half width and cutting length during steady state zone; and (**b**) in depth peak half width profiles in cutting and feed directions for the first point of steady state zone.

The work hardening and defect density gradient beneath the machined surface were evaluated using X-ray peak half width measurement. The peak half width is determined by the distribution of the randomly oriented lattice plane distances. Figure 10a shows the distribution of peak half width in the steady state zone, the results obtained on the same points as in Figure 9. As seen, the peak half width is influenced considerably by the BUE height. The peak half width is reduced when the BUE height increases because of high cutting temperature, which leads to softening of the machined surface and, consequently, reduced peak half width.

Figure 10b shows the in depth evolution of the peak half width in feed and cutting directions for one of the samples in the steady state zone (Point 1). As shown, the highest value of about 1.3° is measured directly at the surface, and this value decreases continuously to 1° for the unaffected workpiece. The depth at which the peak half width stabilizes corresponds to the thickness of the work hardening layer due to machining [6]. For all specimens, this layer was found to be around 250 µm. Peak half width profile was performed on all selected points of the steady state zone and similar results were observed.

### 3.4. Effect of Built-up Edge Formation on Cutting Forces and Chip Formation

**Figure 11:**
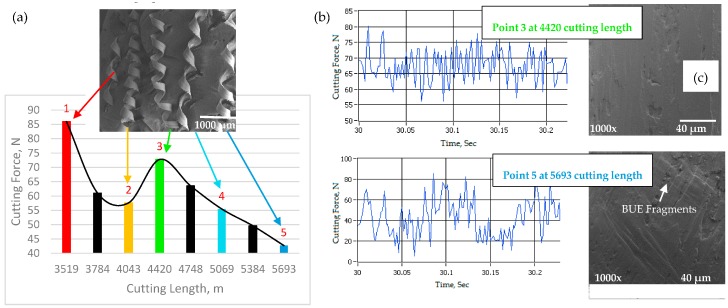
(**a**) Relation between cutting force and cutting length during steady state zone, combined with SEM image of chip formation: and (**b**) cutting force variations; and (**c**) chip undersurface for two selected points in Figure 11a.

To better understand the correlation between the BUE formation and the resulting residual stresses, it was decided to measure the cutting force components while machining the workpiece for the previous discussed residual stress tests. The cutting forces play a significant role in the formation of the residual stresses, although the influence is lower than that of cutting temperature [1]. Figure 11a shows the mean values of the cutting forces in the steady state zone. As shown, as a result of unstable BUE, the cutting force varies greatly, increasing instantly when a BUE is torn off, and decreasing as a new BUE is formed (Figure 5). An increase or decrease in cutting forces may depend on wedge angle; when a BUE increases, the wedge angle is reduced, resulting in a reduction of the work of cutting, cutting temperature and, consequently, cutting force and chip formation [6]. Moreover, the SEM image in Figure 11a shows that the chips formed by the cutting tool with low BUE are curlier than the chips from high BUE, which means that the contact length between the tool and the chip is shorter. Reduced contact length is beneficial in lowering the friction between the chip and the tool [33]. 

It is also observed that the cutting force oscillations are connected with formation and disappearance of BUE: the oscillations are gradually reduced when the BUE is more stable (see Figure 11b). Figure 11c shows SEM images of chip undersurfaces for the two selected points in the steady state zone (Points 3 and 5), they show that the chips formed with large BUE are rougher than those formed with low BUE. As the BUE grows, it becomes unstable and parts of it get removed while cutting. The removed portions of BUE adhere partly to the chip undersurface and partly to the machined surface. This causes the chip undersurface be rough, and friction at the chip–tool interface increases, leading to a reduction the chip speeds during the chip flow and an increase in the area of contact between tool and chip on the rake face surface [34]. 

## 4. Conclusions

The influence of BUE was investigated in wet machining of the austenitic stainless steel-type AISI 304 with the uncoated cemented carbide tool (WC-Co). Specifically, the effect of the variation of BUE height in the steady state zone on process outputs was studied. Surface roughness, residual stresses, and cutting forces were examined to evaluate the BUE formation. The following conclusions can be drawn from this research:

1. BUE formation is an unstable process; it gradually increases layer by layer and when it has reached a certain size, it tears off. This causes cracks and damage on the tool surface, leading to a cutting edge break out. However, sometimes the BUE prevents sliding at the interface, protecting the tool from wear.

2. BUE growth is responsible for a decrease in surface roughness of the machined surface as the BUE is an unstable structure, and, when it is broken off, some of the BUE particles detach from the surface and reduce surface finish. However, in some situations, broken BUE was removed by chips, leading to improved machined surface finish.

3. Tensile residual stresses were found on the surface of the machined workpiece; these stresses were decreasing when the BUE height increased, as the machined surface was softened as a result of high cutting temperature, eventually decreasing the residual stresses. Residual stresses were measured in both feed and cutting directions and it was found that that the hoop residual stresses are larger than the axial component ones, and the maximum compressive stresses and their depth are the same because tool wear remains the same in the steady state zone.

4. The peak half width reduced when the BUE height increased because of high cutting temperature, which leads to softening of the machined surface, causing a reduction in peak half width. Its value was the highest on the surface and then decreased continuously for the unaffected workpiece, whereas the thickness of the work hardening layer was found to be 250 µm.

5. The cutting force varied greatly, increasing when a BUE was torn off, and decreasing as a new BUE was formed. When a BUE increased, the wedge angle was reduced, resulting in a reduction in cutting force. It was also reported that the cutting force oscillations were gradually reduced when the BUE is more stable.

Finally, it is important to emphasize that the BUE formation always follows the same scenario: it forms, accumulates, and breaks off, and then a new BUE is formed again. However, its form and intensity change under same cutting conditions. This study is useful to investigate the beneficial effect of BUE on tool life and understand how the BUE affects the surface integrity and cutting forces.

## Figures and Tables

**Table 1:** Chemical composition and mechanical properties of AISI 304 [29].

Elements	Chemical Composition %	Proof Strength (0.2% Yield) MPa	Tensile Strength MPa	Elongation %	Rockwell Hardness HRC
C	0.08	215	505	70	70
Si	0.75				
Mn	2.0				
P	0.045				
S	0.03				
Cr	20.0				
Ni	0.50				
N	0.10				

**Table 2:** Parameters for X-ray residual stress analysis.

Young’s Modulus (GPa)	Poisson Ratio	Radiation	Bragg Angle (2Φ)	Filter	X ray Elastic Constant ½ S_2_ (MPa^−1^)	X ray Elastic Constant S_1_ (MPa^−1^)
193	0.275	kα	118	Cr	7.036 × 10^−6^	−1.597 × 10^−6^

**Table 3:** EDS analysis of surface on the tool.

Analysis of BUE	Analysis of Insert Surface
Fe% 70.90	W% 94.87
Cr% 29.10	Co% 5.13

**Table 4:** Machined surface and chip undersurface of five selected points in the steady state zone.

Point 1 (3519 m)	Point 2 (4043 m)	Point 3 (4420 m)	Point 4 (5069 m)	Point 5 (5693 m)